# Perception of smile esthetics by laypeople of different ages

**DOI:** 10.1186/s40510-017-0162-4

**Published:** 2017-03-20

**Authors:** Chompunuch Sriphadungporn, Niramol Chamnannidiadha

**Affiliations:** Chulalongkorn University, 34 Henri Dunant Road, Pathumwan, Bangkok 10330 Thailand

**Keywords:** Visual perception, Smile, Esthetics, Age

## Abstract

**Background:**

Age is a factor affecting smile esthetics. Three variables of smile esthetics associated with the maxillary anterior teeth and age-related changes have recently received considerable attention: (i) the incisal edge position of the maxillary central incisors, (ii) the maxillary gingival display, and (iii) the presence of a black triangle between the maxillary central incisors. The aim of this study was to evaluate the influence of age on smile esthetic perception based on these three variables in a group of Thai laypeople.

**Methods:**

The smiles were constructed from a photograph of a female smile. Smile photographs were altered in various increments using three variables: the incisal edge position of the maxillary incisors, gingival display, and a black triangle between the maxillary central incisors. The photographs were shown to a group of 240 Thai laypeople. The subjects were divided into two groups: a younger group, 15–29 years old (*n* = 120) and an older group, 36–52 years old (*n* = 120). Each subject was asked to score the attractiveness of each smile separately using a visual analog scale.

**Results:**

Smile attractiveness scores concerning the incisal edge positions of the maxillary central incisors were similar between the two groups. However, upper lip coverage was rated as unattractive by the younger group. A gingival display of 0 and 2 mm was rated as most attractive by the younger group. Upper lip coverage and gingival display of 0 and 2 mm were considered attractive by the older group. Excessive gingival display (6 mm) was scored as unattractive by both groups. A black triangle ranging from 1 to 2.5 mm between the maxillary central incisors was scored differently between the two groups. The older group was more tolerant of the black triangle size.

**Conclusions:**

Age impacts smile perception based on maxillary gingival display and the presence of a black triangle between the maxillary central incisors, but not of the incisal edge position of the maxillary central incisors. Due to the variation in esthetic perception of each individual, participation between orthodontists and patients for decision-making and treatment planning is a crucial process to provide successful results.

## Background

Recently, the focus on facial esthetics as an indicator of social value has increased. The smile is an important aspect of facial esthetics. Therefore, many orthodontists are incorporating facial esthetics into their treatment planning to achieve a beautiful and youthful smile [1, 2]. However, beauty truly is in the eye of the beholder. The patients’ personal experience and social environment affect their preference towards smile esthetics more highly than the dentists’ or orthodontists’ opinion does [3]. In addition, an individual’s esthetic evaluation is considerably impacted by factors such as education level, social status, and cultural differences [3–5]. Mass media, including television, radio, advertising, movies, magazines, and the internet, also play an important role in the perception of beauty in modern culture [6]. The definition of dental beauty differs across populations, regions, countries, and even continents [5, 7]. Dental beauty is also dynamic, with its parameters changing over time [8]. Currently, the interest in esthetic dentistry has increased, resulting in individuals of different ages seeking orthodontic treatment. Age is a factor affecting the perception of smile esthetics [9]. Previous studies [10–15] have evaluated esthetic perception in terms of smiles with a diastema and midline deviation, smile arc, missing teeth, buccal corridor, and gummy smile in different age groups. The results of most studies [10–13] suggested that there were dissimilar perceptions in different age ranges due to evolving attitudes, lifestyles, and opinions. The maxillary anterior teeth are a key esthetic component of a smile [16]. Three variables of smile esthetics associated with the maxillary anterior teeth and age-related changes have recently received considerable attention: the incisal edge position of the maxillary central incisors [17–19], the maxillary gingival display [20–23], and the presence of a black triangle between the maxillary central incisors [24–26]. As people grow older, these variables can change and may affect smile esthetics. Orthodontists have become increasingly aware of the soft tissue esthetics resulting from treatment and satisfying the patient’s perception of an esthetic outcome [27]. Esthetic treatment planning starts with the position of the maxillary central incisors [28]. The first step in esthetic orthodontic treatment planning is always establishing the vertical position of the maxillary incisors when smiling [29]. The vertical position of the maxillary incisor has great impact on smile esthetics through the smile arc, which been noted by many investigators [14, 29–32]. As a person ages, their smile arc curvature tends to flatten and with worn dentition, a reverse smile arc can develop [33, 34]. Studies have found that the orthodontists’ perceptions and preferences in smile esthetics do not always correlate with those of the patients [19, 35]. However, there was also a study showing similar preferences in esthetics between orthodontists and patients [31]. Therefore, having a thorough knowledge in the perception of this variable may guide orthodontists in preparing an appropriate treatment plan.

Another feature contributing to smile esthetics is the gingival display. Evaluating the amount gingival display in the esthetic zone is crucial [29]. The optimal correlation of the upper lip to the maxillary incisors and gingiva on smiling differed significantly between orthodontists and patients. One ideal upper lip position determined by some studies is for the lower margin of the upper lip to align evenly with the gingival margin of the maxillary central incisors [36, 37]. However, other studies found that some degree of maxillary incisor visibility together with some gingival display is more attractive compared with a complete lack of gingival display or partial tooth coverage by the upper lip [16, 38]. Upper lip coverage tends to increase with age due to lip sagging [39], and therefore, the percentage of gummy smiles may be higher among younger age groups and lower among older adults. Studies of laypeople’s perspectives found a wide range of acceptable gingival display with a maximum of 4 mm of the gingival display and maximum of 4.5 mm of lip coverage, whereas from orthodontists’ point of view, the acceptable range was 0–2 mm [14, 32, 38, 40, 41]. Based on these findings, it is an important error if orthodontists believe that the patients’ esthetic preferences are the same as theirs.

Another parameter affecting the perception of an esthetic smile is the presence of black triangle, which arises from decreased papilla length at the contact point between the central incisors [42], resulting in the embrasure cervical to the interproximal contact not being filled by gingival tissue [43]. Anatomically, this is a minor issue; however, from an esthetic viewpoint, this small space is of great importance, especially in the anterior teeth, because it is quite visible when smiling [44, 45]. A space between the incisors due to loss of the interdental papilla and bone is more common in adult patients [46], occurring in more than 1/3 of adults [47]. Moreover, this space is also common in post-orthodontic treatment, found in 38 and 42% of adult [47] and adolescent [42] patients, respectively. Understanding a patient’s preference prior to commencing therapy may help in developing an appropriate treatment plan and successful result.

Previous studies [10–15] found disparate results when evaluating the relationship between age and smile perception in various aspects such as smile arc, gingival display, midline diastema, missing teeth, and a black triangle between the maxillary central incisor and buccal corridor. Lacerda-Santos et al. [12] compared the smile attractiveness of the various sized buccal corridors between groups of individuals in different age ranges. Laypeople over 65 years old were found to be less critical when evaluating the different smile images compared with the younger group. In addition, Gerritsen et al. [13] found that Tanzanian subjects over 45 years old were less dissatisfied with missing maxillary teeth compared with those lower than 45 years old. In contrast, Gracco et al. [15] found that there was no significant difference in assessment of the esthetic value of the buccal corridor on smile perception between age groups. Another feature related with smile perception is the black triangle. The presence of a black space between the maxillary central incisors was considered more attractive by the older group compared with the younger group [11]. When assessing gingival display by Mokhtar et al. [10], the older group (over 40 years old) was less tolerant than the younger group. Moreover, the presence of a diastema was more accepted in older group than younger group. In contrast, a study [14] considering the influence of the smile arc in conjunction with gingival display on smile attractiveness found that age had no effect on esthetic perception.

The majority of these studies [10–13] found a relationship between age groups and the perception of a smile. However, the influence of age on smile perception remains unresolved [10–12, 14]. The null hypothesis in our study was that differences in these variables would be rated as equally attractive by different age groups. Therefore, the aim of this study was to evaluate the influence of age on smile esthetic perception, using varying incisal edge positions of the maxillary central incisors, maxillary gingival display, and the presence of a black triangle between the maxillary central incisors.

## Methods

This study was approved by the Human Research Ethics Committee of the Faculty of Dentistry, Chulalongkorn University. Based on the results of a pilot study, a sample size calculation was performed using n4studies (Version 1.4.1) [48]. Using a significance level of alpha = 0.05 and the sample size was calculated to achieve 80% power. The sample size calculation indicated that 95 subjects were needed in each group.

The subjects were selected by purposive sampling. Two-hundred and forty Thai laypeople living in Bangkok, Thailand, were asked to participate in this study. The subjects were categorized into two groups based on their generation: generation Y: 15–29 years old (*n* = 120) and generation X: 36–52 years old (*n* = 120), as defined by Strauss and Howe [49, 50]. Each age group was comprised of 50% males and females. The participants were recruited from shopping malls, educational institutions, and offices. Dental professionals and dental students were excluded from this study.

### Photo album

The photo album used for evaluation consisted of three photo sets based on three variables: incisal edge position of the maxillary incisors, gingival display, and black triangle. Each set included six different photographs and one randomly selected repeated smile photograph to test reliability. The selected smile was a frontal view of a young adult Thai female. To minimize any distracting variables, other facial structures were excluded from the smile photographs. The smile features in the photographs were digitally altered into 18 photographs using Adobe Photoshop CS6 (Adobe Systems Inc., San Jose, CA). The photographs were manipulated to create a symmetrical image and adjusted using a ruler (present in the photograph) to represent the actual size of the patient’s teeth. The modifications were intentionally created to demonstrate a smile esthetic discrepancy. The photographs were grouped into three sets, each representing an altered smile feature in various increments. The alterations were chosen following consultation with clinically experienced orthodontists and adopted from previous studies [11, 19, 35, 40].

#### Set 1: the incisal edge position of the maxillary central incisors

**Fig. 1:**
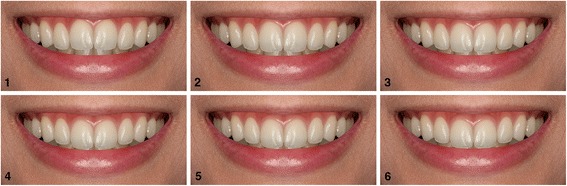
Altered incisal edge position of the maxillary central incisors in 0.5-mm increments. **1** 0.5 mm intruded; **2** 0 mm extruded; **3** 0.5 mm extruded; **4** 1.0 mm extruded; **5** 1.5 mm extruded; and **6** 2.0 mm extruded

Gingival margins of the central incisors and canines were equal, and the incisal edges of the central incisors were 0.5 mm inferior to the lateral incisors in the reference image. The incisal edge position of the maxillary central incisors was adjusted incisally or gingivally using 0.5-mm increments, with the line between the gingival margin of the central incisors and canines serving as a reference plane. The maxillary central incisors were moved gingivally and incisally 0.5 and 0.5–2.0 mm, respectively (Table 1 and Fig. 1).

**Table 1:** Characteristics of the smiles used in this study

Adjusted vertical positions of the maxillary central incisors	Gingival margins of the maxillary central incisors	Central to lateral incisor edge level
(1) 0.5 mm intruded	0.5 mm above the canines	0 mm
(2) 0 mm unaltered	Equal with the canines	0.5 mm
(3) 0.5 mm extruded	0.5 mm below the canines	1.0 mm
(4) 1.0 mm extruded	1.0 mm below the canines	1.5 mm
(5) 1.5 mm extruded	1.5 mm below the canines	2.0 mm
(6) 2.0 mm extruded	2.0 mm below the canines	2.5 mm

#### Set 2: gingival display

**Fig. 2:**
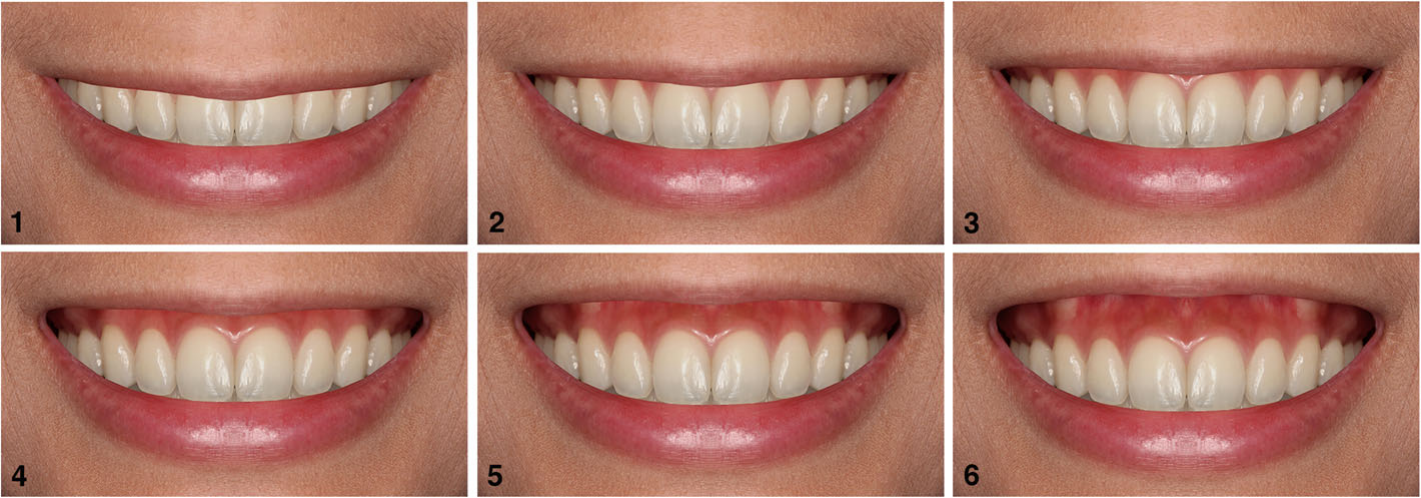
Altered gingival display in 2.0-mm increments. **1** −4 mm; **2** −2.0 mm; **3** 0 mm; **4** +2.0 mm; **5** +4.0 mm; and **6** +6 mm

The distance between the upper lip and gingival margin of the maxillary incisors was 0 mm in the reference image. The gingival display was altered using 2-mm increments by decreasing (−) the distance of the gingival margin between the maxillary incisors and upper lip by 2.0 and 4.0 mm and by increasing (+) the margin by 2.0, 4.0, and 6.0 mm (Fig. 2).

#### Set 3: black triangle between the maxillary central incisors

**Fig. 3:**
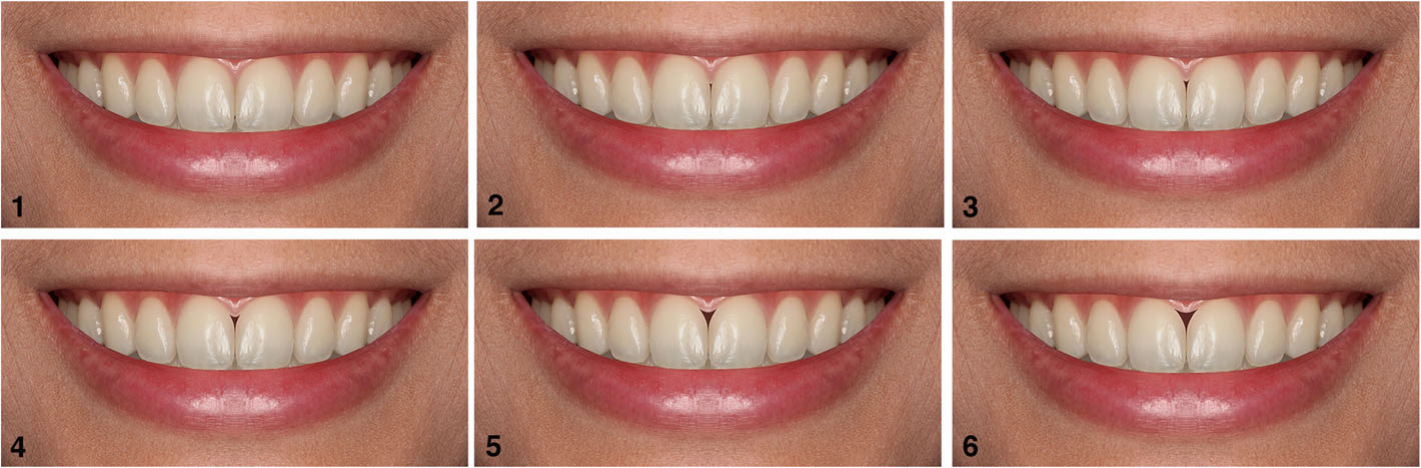
Altered black triangles between the maxillary central incisors in 0.5-increments. **1** no black triangle; **2** 0.5 mm; **3** 1.0 mm; **4** 1.5 mm; **5** 2.0 mm; and **6** 2.5 mm

Black triangles of different sizes were created between the maxillary central incisors. This resulted in six photographs: the reference image with no black triangles and the other images with increasing sizes of black triangles, using 0.5-mm increments (0.5, 1, 1.5, 2, and 2.5 mm) (Fig. 3).

The photographs were printed on photographic paper to create a photo album. The photographs in each set were coded from 1 to 6. Photograph number 7 was identical to one randomly selected image in the set to assess reliability. The sets of photos were arranged according to set number; however, the photographs displaying the incremental changes were randomly arranged.

### Questionnaires

Questionnaires were distributed to the participants. The participants were asked to score the attractiveness of each smile image separately using a visual analog scale (VAS), graded from least attractive to most attractive. The questionnaire consisted of two parts:

1. Part 1 elicited demographic data of the participant to determine nationality, sex, and age.
2. Part 2 consisted of visual analog scales pages.

A 10-cm VAS was used for individual ratings. The left end (at zero) of the scale was labeled as least attractive and the right end (at the 10-cm range) was labeled as most attractive. Each participant was asked to mark along the VAS according to their perception of dental esthetics. Each mark on the VAS was measured with a caliper and recorded. The participants were requested to not directly compare the images. The time to evaluate each image was limited to 1 min.

### Statistical analysis

The data was found to be not normally distributed using the Kolmogorov–Smirnov test. The differences in scores between photographs within a given set were evaluated using Friedman’s test and the Wilcoxon signed-rank test for pair-wise comparisons. The differences in scoring between age groups were assessed using the Mann-Whitney *U* test. All tests were performed at a 0.05 significance level. To assess intraparticipant agreement, the intraclass correlation coefficient (ICC) was used to compare image scores.

## Results

The mean ages in the 15–29 year-old and 36–52 year-old groups were 22 and 43, respectively. The intraclass correlation coefficients for both participant groups were equal to or higher than 0.79, which indicated good reliability [51].

There was no significant difference in esthetic scores between the male and female participants in either age group. Therefore, the data for the male and female participants in each age group were pooled and used for further analysis.

### The incisal edge position of the maxillary central incisors

There was no significant difference in scoring between the images either within or between age groups (Table 2).

**Table 2:** Photograph ratings by age group of altered incisal edge positions of the maxillary central incisors

Central-lateral incisor edge level (mm)	Age groups						*p* value
	15–29 years old			36–52 years old			
	Mean	SD	Result^a^	Mean	SD	Result^a^	
Image 1 (0)	6.80	1.88	A	6.69	1.67	A	0.454 NS
Image 2 (0.5)	6.90	1.57	A	6.96	1.65	A	0.322 NS
Image 3 (1)	6.98	1.80	A	6.77	1.63	A	0.192 NS
Image 4 (1.5)	7.04	1.72	A	6.78	1.54	A	0.132 NS
Image 5 (2)	6.74	1.85	A	6.79	1.87	A	0.964 NS
Image 6 (2.5)	6.89	1.92	A	6.83	1.76	A	0.549 NS

*NS* not significant

^a^In each age group, scores of the images with the same letter were not significantly different

### Gingival display

In the younger group, −4 and +6 mm gingival displays were rated as the least attractive, whereas 0 and +2 mm gingival displays were perceived as the most attractive (Table 3). In the older group, deviations from −4 to +2 mm had no significant effect on scores, while a +6 mm gingival display was perceived as the least attractive. Comparison between age groups showed a significant difference for −4 to +2 mm gingival displays (*p* < 0.05). The younger age group gave lower ratings for gingival displays of −4 and −2 mm compared with the older group. In contrast, 0 and +2 mm gingival displays were given higher ratings in the younger group compared with those in the older group. However, excessive gingival display (+6 mm) was rated as the most unattractive in both groups.

**Table 3:** Photograph ratings by age group of altered gingival display

Gingival display (mm)	Age groups						*p* value
	15–29 years old			36–52 years old			
	Mean	SD	Result^a^	Mean	SD	Result^a^	
Image 1 (−4)	2.93	1.67	A	5.90	1.96	A	0.000**
Image 2 (−2)	3.86	1.38	B	5.89	1.80	A	0.000**
Image 3 (0)	6.88	1.42	C	5.81	1.61	A	0.000**
Image 4 (+2)	7.40	1.36	C	5.78	1.59	A	0.000**
Image 5 (+4)	4.74	1.71	B	4.64	1.46	B	0.532 NS
Image 6 (+6)	2.98	1.96	A	2.94	1.49	C	0.882 NS

*NS* not significant

^**^Statistical differences between age groups (*p* < 0.05)

^a^In each age group, scores of the images with the same letter were not significantly different

### Black triangle between the maxillary central incisors

Evaluation of a black triangle tended to result in a lower score as the size of the space increased in both age groups; however, the older group gave higher scores compared with the younger group at the same space size (Table 4). The images without a black triangle were rated as the most attractive, whereas the lowest scores were seen for the 2- and 2.5-mm black triangle images. Compared between age groups, there were no significant differences in scores of the absence of a black triangle and a small black triangle (0.5 mm). Increasing the size of the black triangles from 1 to 2.5 mm resulted in significant differences between the age groups; with the older group being more tolerant of the size of black triangles (*p* < 0.05).

**Table 4:** Photograph ratings by age group of altered black triangles

Black triangle (mm)	Age groups						*p* value
	15–29 years old			36–52 years old			
	Mean	SD	Result^a^	Mean	SD	Result^a^	
Image 1 (0)	7.05	2.23	A	7.24	1.79	A	0.805 NS
Image 2 (0.5)	5.53	2.46	B	6.13	1.86	B	0.058 NS
Image 3 (1)	4.59	2.22	C	5.36	1.89	C	0.002**
Image 4 (1.5)	4.28	2.22	C	5.02	2.00	C	0.002**
Image 5 (2)	3.38	2.25	D	4.32	2.00	D	0.000**
Image 6 (2.5)	2.98	2.26	D	3.87	2.07	D	0.001**

*NS* not significant

^**^Statistical differences between age groups (*p* < 0.05)

^a^In each age group, scores of the images with the same letter were not significantly different

## Discussion

This study evaluated the differences in smile esthetic perception between a younger and older age group. Our results indicated the presence of differences in perception between these groups. Based on these findings, the null hypothesis was rejected. Our study is the first to demonstrate the perception of the vertical position of the maxillary central incisors when smiling by different aged laypeople. We found no statistical difference in the participants’ preference for vertical incisal edge position. Comparing the two age groups, age did not affect the perception of smiles when varying this variable. Both groups shared similar preferences when evaluating minor discrepancies in incisal edge positions of the maxillary central incisors at any level. This result was inconsistent with the study of Machado et al. [19], who showed a preference for having a different vertical edge position between the central and lateral incisors among college students. The most attractive smiles for laypeople were smiles with a 1–2-mm central to lateral incisor edge level difference, whereas a study performed by King et al. [52] noted this step was only 0.6 mm. These differences with our findings are probably because laypeople are not as sensitive to such minor discrepancies as orthodontists, as shown by Machado et al. [19]. This study found that laypeople were more tolerant of minor discrepancies by ranking altered smiles with higher scores. King et al. [52] also stated that orthodontists had a smaller range of acceptable altered maxillary central to lateral incisor edge levels compared with laypeople. Furthermore, in our study, it is possible that some participants liked both flat and consonant smiles.

The results of our study imply that minor discrepancies between maxillary central and lateral incisor edges have no influence on laypeople’s perception. This is probably because the discrepancies were symmetrical, as asymmetrical discrepancies strongly affected their perception in a previous study [35, 53]. Even a slight incisal edge discrepancy of 0.5 mm between the maxillary central incisors was considered as unattractive by laypeople and orthodontists [35]. This may indicate that as long as the maxillary central incisors are symmetrical, minor vertical position differences between the maxillary central and lateral incisors do not always need to be treated. Thus, orthodontists should not make these decisions alone; the patients should also participate to establish an appropriate treatment plan, as many studies [19, 35, 38, 40, 41, 53] concluded that orthodontists are more observant in detecting deviations from ideal positions. Thus, their decisions might be based on excessive concern and lead to unnecessary treatment.

When considering the maxillary gingival display, a disagreement in smile attractiveness was found between age groups. The younger group rated a gingival display of 0–2 mm as the most attractive. This finding corresponds with the study of Hunt et al. [54] and Geron et al. [55], with the latter stating that some gingival display is often esthetically appealing because it corresponds with a more youthful appearance.

The tendency in the younger group was that increased upper lip coverage of the teeth resulted in a more unattractive smile. This perception was probably caused by the assumption that upper lip coverage is a sign of aging [39]. In the older group, upper lip coverage was preferred, as this is prone to occur at their age. These different esthetic perceptions might be explained by the form concept [56], which states that the more an individual experiences certain smile appearances, the more likely they are to perceive it as being normal. Another possible reason was that adults’ teeth tend to have more defects such as black triangles, spacing, crowding, or restorations. Excessive tooth exposure may thus reveal what they would rather conceal. These findings for the younger group are inconsistent with those of Ioi et al. [5] who reported that young laypeople preferred a smile with tooth coverage by the upper lip. These findings might be related to ethnic and social differences in smile preference.

Although gummy smiles may be more common among younger age groups [57] and less common among older adults [58], it is noteworthy that excessive gingival display (6 mm) was not tolerated by either age group. Increasing gingival display from 4 to 6 mm significantly impacted attractiveness, with rating scores decreasing by 37%. These results coincided with those of Kokich et al. [40] and Ker et al. [7], which noted that laypeople were tolerant of a gummy smile up to 4 mm.

Ioi at al. [5] reported that both Asian adolescences and adults showed a threshold of acceptability for upper incisor coverage of 0–5 mm in males and 0–2 mm in females. In contrast, our study found that adults accepted upper lip coverage as well as a gummy smile of up to 4 mm. In contrast, adolescents and young adults rated upper lip coverage as unattractive at any level. The perception of a pleasing smile remains individually subjective and culturally dependent.

Multiple [16, 36, 37, 54] suggest that the ideal upper lip position when smiling should align evenly or deviate up to 2 mm from the gingival margin of the upper incisors. Thus, orthodontists tend to treat patients from a more academic perspective rather than a subjective one. Studies have shown that orthodontists are more sensitive to a gummy smile compared with laypeople [14, 40, 59]. To ensure patient satisfaction with treatment results, making a joint decision between the orthodontist and the patient before the start of the treatment is crucial.

The results of both groups concerning the esthetic effect of black triangles between the maxillary central incisors were similar, i.e., the larger the black triangle, the lower the images were rated. Thus, it is not surprising that in both groups, the absence of a black triangle was considered the most pleasing. However, the presence of a very small space (0.5 mm) was likely acceptable because it is too small to affect the perception of laypeople at any age.

Pithon et al. [11] found no significant difference between black space esthetic scores in groups 15–19 and 35–44 years old. However, in our study, the score of images presenting a 1–2.5-mm black triangle was significantly different between age groups. The older group gave higher scores for all images. These findings indicate that the older group was more tolerant of having a black triangle compared with the younger group. This is probably because black triangles are more common in the adult population, as aging leads to a reduction in interdental papilla height [60]. Thus, older individuals are likely to be more tolerant of this appearance.

We assumed that esthetic scores ranging from 0 to 5 denoted unattractive smiles and scores higher than 5 denoted attractive smiles. Based on this assumption, a 0.5-mm black triangle represented the threshold of acceptability in the younger group, whereas in the older group, 1.5 mm was the limit of acceptability. The presence of 2–2.5-mm black triangles resulted in the lowest scores in both groups. These results indicated that the participants in our study were slightly more sensitive to black triangles compared with laypeople in the study of Kokich et al. [40], who found that laypeople could detect a 3-mm open gingival embrasure. This difference may reflect that esthetic perception gradually changes over time. However, the patient must be informed prior to treatment of the possibility of this space being created at the end of orthodontic treatment, and the orthodontist should avoid creating this defect. To avoid unnecessary treatment, small discrepancies could be left in some cases, particularly in older patients.

In addition to the presence of a black triangle between the upper central incisors affecting the smile perception, black triangles can be found between other anterior teeth, which also might affect the esthetics. A comparison of these variables is suggested in future studies.

## Conclusions

Age has an impact on the perception of smile esthetics in terms of maxillary gingival display and the presence of a black triangle between the maxillary central incisors, but not of the incisal edge position of the maxillary central incisors. An ideal smile based on academic considerations may not be perceived as the most attractive by laypeople. Due to the variation in esthetic perception by each person, participation between orthodontists and patients for decision-making and treatment planning is crucial to generate successful results.

## Abbreviations

ANOVA: Analysis of variance
ICC: Intraclass correlation coefficient
VAS: Visual analog scale
